# Education of Patients With Atrial Fibrillation and Evaluation of the Efficacy of a Mobile Virtual Patient Environment: Protocol for a Multicenter Pseudorandomized Controlled Trial

**DOI:** 10.2196/45946

**Published:** 2024-01-23

**Authors:** Panagiotis Antoniou, Eleni Dafli, George Giannakoulas, Gaukhar Igimbayeva, Olga Visternichan, Serhii Kyselov, Ivetta Lykhasenko, Dmytro Lashkul, Ilia Nadareishvili, Sergo Tabagari, Panagiotis D Bamidis

**Affiliations:** 1 Lab of Medical Physics and Digital Innovation School of Medicine Aristotle University of Thessaloniki Thessaloniki Greece; 2 1st Cardiology Department AHEPA University General Hospital of Thessaloniki Thessaloniki Greece; 3 Orhun Medical Clinic Almaty Kazakhstan; 4 Internal Medicine Department Karaganda Medical University Karaganda Kazakhstan; 5 Department of Internal Diseases No.1 and Simulation Medicine Zaporizhzhia State Medical University Zaporizhzhia Ukraine; 6 Department of Propedeutics of Internal Diseases, Radiation Diagnostics and Radiation Zaporizhzhia State Medical University Zaporizhzhia Ukraine; 7 AIETI Medical School David Tvildiani Medical University Tbilisi Georgia

**Keywords:** atrial fibrillation, virtual patient, scenario based learning, technology enhanced learning, mHealth, mobile health, patient engagement, patient education, cardiac arrhythmia, mortality, mobile application, mobile app, health education, randomized control trial, cardiology, cardiac, heart, Greece, Ukraine, Kazakhstan, clinical decision support systems, CDSS, virtual patient scenario, myocardial infarction, arrhythmia, stroke

## Abstract

**Background:**

Atrial fibrillation (AF) is the most common sustained cardiac arrhythmia and is a leading cause of mortality and morbidity. Patient knowledge about AF and its management is paramount but often limited. Patients need to be appropriately informed about treatment options, medicinal adherence, and potential consequences of nonadherence, while also understanding treatment goals and expectations from it. Mobile health apps have experienced an explosion both in their availability and acceptance as “soft interventions” for patient engagement and education; however, the prolific nature of such solutions revealed a gap in the evidence base regarding their efficacy and impact. Virtual patients (VPs), interactive computer simulations, have been used as learning activities in modern health care education. VPs demonstrably improved cognitive and behavioral skills; hence, they have been effectively implemented across undergraduate and postgraduate curricula. However, their application in patient education has been rather limited so far.

**Objective:**

This work aims to implement and evaluate the efficacy of a mobile-deployed VP regimen for the education and engagement of patients with AF on crucial topics regarding their condition. A mobile VP app is being developed with the goal of each VP being a simple scenario with a set goal and very specific messages and will be subsequently attempted and evaluated.

**Methods:**

A mobile VP player app is being developed so as to be used for the design of 3 educational scenarios for AF management. A pseudorandomized controlled trial for the efficacy of VPs is planned to be executed at 3 sites in Greece, Ukraine, and Kazakhstan for patients with AF. The Welch *t* test will be used to demonstrate the performance of patients’ evaluation of the VP experience.

**Results:**

Our study is at the development stage. A preliminary study regarding the system’s development and feasibility was initiated in December 2022. The results of our study are expected to be available in 2024 or when the needed sample size is achieved.

**Conclusions:**

This study aims to evaluate and demonstrate the first significant evidence for the value of VP resources in outreach and training endeavors for empowering and patients with AF and fostering healthy habits among them.

**International Registered Report Identifier (IRRID):**

PRR1-10.2196/45946

## Introduction

### Impact of Atrial Fibrillation and Non–Vitamin K Oral Anticoagulants and the Role of Integrated Management

Atrial fibrillation (AF) is the most frequent long-term cardiac arrhythmia in adults, presenting a considerable burden on patients, health care systems, societal health, and the global health economy [1]. Because AF is associated with significant morbidity and mortality, numerous research efforts and resources are being directed toward gaining more detailed information about the mechanisms underlying AF, its natural course, and effective treatments, and new evidence is being generated and published on a regular basis [2].

The current estimated prevalence of AF is between 2% and 4% [3], with a 2.3-fold increase projected due to the general population's increased longevity and the intensive search for AF [4]. AF is a well-known risk factor for thrombus development in the left atrium and eventual embolism on the left side. AF raises the risk of stroke by 5 folds, but the risk varies depending on the presence of stroke risk factors or modifiers [2]. Oral anticoagulants (OAC) are the cornerstone of AF treatment. Vitamin K antagonist (VKA) medication (mainly warfarin) lowers the risk of stroke and death by 64% and 26%, respectively, when compared to control or placebo [5]. Non–vitamin K oral OACs (NOACs) have outperformed VKAs in most therapeutic circumstances. NOAC medications do not have the practical constraints of VKAs, such as a small therapeutic window, interactions with food and other treatments, and the need to monitor coagulation levels. In 4 large randomized controlled trials (RCTs) with patients with AF, NOACs were compared to warfarin [6-9]. They were demonstrated to be at least noninferior to VKA therapy for the prevention of stroke or recurrent venous thromboembolism and were associated with a lower risk of bleeding. In a meta-analysis of these RCTs, NOACs were associated with a 19% significant reduction in the risk of stroke or systemic embolism, a 51% reduction in the risk of hemorrhagic stroke, and a similar reduction in the risk of ischemic stroke compared to VKAs. NOACs are also associated with a 10% reduction in all-cause mortality [10].

To provide optimal medical treatment to patients with AF, integrated management necessitates a patient-individualized care route. Treatment choices should be reviewed and a management plan agreed upon with health care experts in this patient-centered approach [11]. Treatment is liable to alter over time as new symptoms, risk factors, disease progression, and novel medicines emerge. To prevent stroke and improve symptoms, it is critical to consider optimizing resource usage. The initial stage in shared decision-making should be to investigate the patients' preferences [12]. The importance of stroke prevention and rhythm control among patients, as well as the corresponding risks of death, stroke, and significant bleeding, should be properly appraised for shared decision-making.

Patients’ awareness of AF and its management is sometimes restricted, especially when first diagnosed, because the majority of treatment decisions must be addressed and made. Furthermore, controlling patients' expectations of treatment goals, as well as ensuring that patients are correctly informed about treatment options, how to better adhere to therapy, and potential repercussions of nonadherence, are critical to increase adherence [2]. Thorough education of physicians on these approaches, as well as correct adherence and active engagement of the patient in the treatment process, are critical to the success of each treatment plan. Soft health interventions for education and empowerment of both clinicians and patients are critical in this environment.

### Education and Other Soft Interventions Involving Mobile Health Apps in Cardiology

There has been an explosion of mobile health (mHealth) apps during the previous decade, with an estimated 3.7 billion downloads globally between 2013 and 2017 [13], many of which are intended for AF. A 2020 systematic review revealed around 11,152 articles related to mHealth apps for AF but only included 9 studies with real outcomes about mHealth therapies for AF in its results synthesis [14]. This indicates the prevalence of such solutions, as well as the dearth of data on their efficacy and impact. Only a few apps have been evaluated formally [15-17]. It should be emphasized that there are few apps that are specifically intended for patients. There are numerous informative apps that patients can use; however, a preferred option would be the development of a specific app for the treatment of a given ailment in order to help afflicted individuals in a more appropriate way [18].

Clinical decision support systems that digitize and provide evidence-based recommendations, therapeutic pathways, and algorithms to facilitate individualized, timely, and evidence-based treatment could be a valuable aid in the holistic management of AF. To improve patient-integrated AF management [19], the MobiGuide project [20] and numerous applications have been deployed. The European Society of Cardiology–Characterizing Atrial fibrillation by Translating its Causes into Health Modifiers in the Elderly Consortium partnership offers a smartphone or tablet app for patients with AF; however, it has yet to be tested prospectively [21]. Contradictory findings highlight the need for more properly designed trials, including evaluation of the intervention's influence on clinical outcomes [22].

As a result, the scope and impact of mHealth apps for AF, as well as the level of patient and health care professional (HCP) engagement and acceptability, are currently unknown. HCP engagement refers to information sharing between the patient and provider, shared responsibilities in decision-making processes, and support of patients’ choices and acceptability to a degree to which an intervention is approved by most HCPs. Given both patients and HCPs may easily use these applications, it is critical to understand their scope and content and their acceptability among users, and to investigate the purpose and results of app adoption and usage.

### Virtual Patients in Health Care Education

Virtual patients (VPs)—interactive computer-based clinical scenarios for the purpose of medical training, education, or assessment—have been increasingly used as learning activities in current health care education, particularly in teaching decision-making through scenarios [23]. Because VPs can enhance cognitive and behavioral skills, they have been successfully incorporated in undergraduate and postgraduate curricula [24]. With the rising use of VPs, there are opportunities for pedagogical synergies to allow trainees of diverse categories to practice in realistic and safe learning contexts [25]. The key characteristics of VP systems are that they enable repetitive and intentional practice by any student, regardless of time or physical location, and that mistakes are not fatal. These opportunities provided by VPs in current medical education, combined with positive evaluation results from various studies demonstrating that they may improve cognitive and behavioral skills better than traditional methods [24], have resulted in a widespread trend toward VP creation and use among academic institutions [26]. Furthermore, VPs enable the production and usage of more game-based educational content, which provides the student more exploratory flexibility and provides a different area for case-based content in current medical education.

The widespread adoption of these digital technologies, not only in medical student education but also in the health care community in general and in the patient community, has undoubtedly been limited thus far, but it has the potential to educate and psychologically support high-risk patients and vulnerable populations in these new and unprecedented circumstances. Because numerous academic institutions have the VP resources and expertise in their execution, such effective educational content might be simply repurposed in a more patient-centered format and be used more widely by the patient community as an educational aid.

### Study Aim and Objectives

In this technological setting, we propose the development of a holistic approach to patient engagement and education based on the fundamentals of AF. The DEEP-RAFT (Doctors’ Education, Empowerment of Patients, Regarding Atrial Fibrillation and Venous Thromboembolism) project would generate a suite of educational and informational interventions along an axis created by 2 poles: digital content cocreation and evidence-based educational impact. This effort will be based on a suite of digital teaching resources, in the form of VPs, co-designed by continuous and immediate involvement of health care specialists, health care policy influencers, and patients. This strategy seeks, first and foremost, to develop materials that are more patient-centered and address realistic problems relevant to the health care systems involved in the project. The focus of this effort is on the second topic, evidence-based educational impact, which attempts to demonstrate the educational efficacy and acceptance of the generated resources as they may be used in a diverse but targeted set of education and outreach activities.

In practice, a mobile app for natively deploying VPs on mobile devices is developed, along with a list of relevant VPs. In the following parts, we will provide these concrete and intangible tools and approaches to contextualize the protocol that is described below.

## Methods

### Study Design

This is a 2-arm, parallel-group pseudorandomized controlled trial that will be performed at 3 sites—Greece, Ukraine, and Kazakhstan—and will include the evaluation of the educational VP interventions. This will be conducted in 2 axes that correspond to the primary outcomes of the study. The first axis aims to determine the efficacy of the educational VP interventions, while the other will involve assessing the acceptance and opinions of the participants about the VP modality for education and information purposes. Correspondingly, the primary outcomes of the study include the efficacy and the acceptance of the VP interventions, and the secondary outcomes include the opinions of the participants about the VP modality for education and information purposes. The aims of the study are summarized in the following research question: are patients with AF better educated about their post–acute phase lifestyle changes and needs by using mobile virtual scenarios compared to conventional patient education methods?

Α prepilot arm of the study will be initially conducted in Greece and will consist of hospitalized patients due to AF episodes, all of which should complete a short quiz. From among the patients who complete the quiz, half of the patients will be randomly allocated to the control (normal clinical information) cohort, while the other half will be informed with the help of VP vignettes.

After the prepilot arm, the pseudorandomized controlled trial will be conducted multicentrically. The same process, as in the prepilot arm, will be followed for recruiting the core sample, exploring the efficacy and impact of VPs in educating patients with AF. Since this trial will have been conducted during the COVID-19 pandemic, all national and international health and safety protocols will be followed.

### Inclusion and Exclusion Criteria and the Recruitment Process

The inclusion and exclusion criteria are outlined in Textbox 1. The dropout criterion is withdrawing consent during the study period.

In the Greek pilot where randomization of patient cohorts would occur, a simple coin toss algorithm will be used, implemented by Excel’s (Microsoft Corp) RANDBETWEEN function. This will allocate patients between the VP education cohort and the control (normal clinical information) cohort. In the Ukraine pilot, a sampling of convenience will allocate most of the participants to the VP cohorts and fewer to the control (normal clinical information) cohort. In the Kazakhstan pilot, all accepted patients will be allocated to the VP intervention cohort. While this decision by the medical team of this center makes impossible the conduct of a distinct local evaluation of impact, we chose to include the sample in the multicentric data processing part of the study. It should be noted that evaluation results will be extracted on a per-site basis only in the Greece and Ukraine arms of the study. In the multisite comparison, the whole sample of intervention patients (including the totality of the Kazakh cohort) will be compared to the totality of the sample of control patients from Greece and Ukraine cohorts.

Patients who meet the requirements for that study will be informed about the study and will be asked if they would like to participate in it. They will then sign the consent form to take part in the study. Patients who may withdraw their consent during the study will be excluded from the analysis.

**Study inclusion and exclusion criteria.**

**Inclusion criteria:**

- Patients with a history of paroxysmal, persistent, or permanent atrial fibrillation
- Access to an internet connection and adequate equipment
- Mastery of the country’s first language
- Informed consent provided by the participant

**Exclusion criteria:**

- Organic or symptomatic mental disorders
- Alzheimer disease
- Mental and behavioral disorders due to alcohol abuse
- Mental and behavioral disorders due to drug abuse
- Refusal of patients to receive basic drug therapy

### Intervention Cohorts

**Figure 1:**
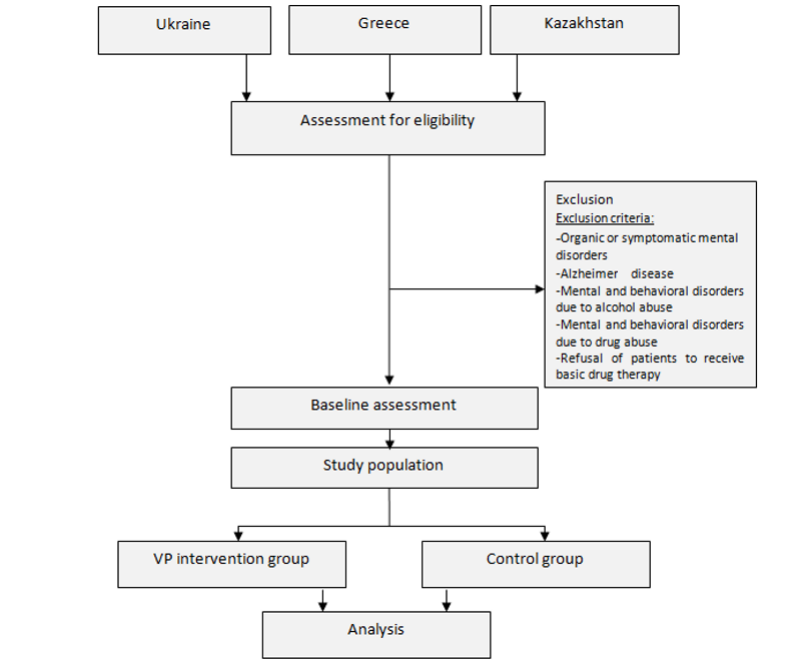
The flowchart of the full pilot arm of the study. VP: virtual patient.

The full pilot arm of the study will be conducted multinationally in Greece, Ukraine, and Kazakhstan (Figure 1). In Greece, a pool of 700 patients will be reached by phone, and they will participate in the trial from their home. From among the patients contacted remotely, and from those who respond, those who would be eligible will be asked to complete the intervention. From among the patients who choose to complete the quiz, half of them will be randomly allocated to the control (normal clinical information) cohort, while the other half will be informed with the help of VP vignettes. In Ukraine, 160 patients will be reached and will be divided into the control and intervention groups. Finally, at the Kazakhstan center, 190 patients will be contacted.

Given that all recruitment and adherence criteria are followed, sufficient sample sizes for control and intervention groups will be ensured so as to use the Welch *t* test for analysis, which is robust to large sample size inequalities, in order to not exclude any useful data from the generalized multicenter VP cohort.

### Blinding (Masking)

Participants and trial personnel will not be blinded after the point of assignment to interventions because of the nature of the interventions and outcomes assessed. Participants will know which group they will belong to because the group-specific intervention will follow immediately after randomization.

### The Intervention

The 2 groups that will be compared are as follows.

#### The VP Cohort

In this cohort, doctors of the research team will contact patients and ask them to participate in the VP vignette case (see *The VPs* section for a detailed description of the virtual scenarios used in the intervention, and the *The Technical Architecture of the Mobile App* section for details of the mobile app that will be used for delivering the intervention). At the end of the case questions, further information will be provided.

#### The Control (Normal Clinical Information) Cohort

In this cohort, doctors of the research team will contact patients and provide the protocol—this will include dictated information for patients with AF.

### The Technical Architecture of the Mobile App

A mobile VP player app has been created, which is capable of tracking the following user data: a detailed log of pathway detection and tracking, time spent in each node of the VPs or mobile VP, tracking of milestones, rate of successful completion of the VPs or mobile VP, and connection to learning outcomes. The player follows a flat development approach and should be available as a progressive web application. A progressive web application is a type of web-based application software that is built using standard web technologies such as HTML, CSS, and JavaScript. It is aimed to be compatible with any platform that supports a standards-compliant browser. The ability to work offline, receive push notifications, and access device hardware will enable the creation of user experiences similar to those found in native applications on desktop and mobile devices. Because a progressive web application is a subset of a web page or website known to be a web application, neither developers nor users should be required to install web applications through digital distribution systems such as the Apple App Store or Google Play.

Technically, communication between the backend and the front end player is accomplished through the use of the JSON application programming interface standard [27], which is an extremely efficient method of exchanging data over slow networks (eg, mobile phone networks).

The system's appearance and feel are based on Bootstrap, a free and open-source CSS framework for developing responsive, mobile-first front end web applications. It would be optimized for smartphone screens so as to provide a “mobile-first” user experience.

### The VPs

Three scenarios will be explored by the participating medical teams. All will be focused on the patients, since these are the target group of the VPs. A tabulated outline of these scenarios is presented in Table 1. The first scenario should be dealing with a chronic case of AF, including guidance for good medication adherence, systematic medical appointments, and correct communication. The second scenario would be about detecting early onset and prevention of paroxysmic AF. The third scenario will deal with a case of AF that involves lifestyle-compounding factors to the disease (smoking, drinking, and unhealthy eating), as well as more heavy complications that are significantly more probable in such cases.

**Table 1:** Outline of suggested scenarios and educational objectives of virtual patients.

Scenario theme	Educational objectives (after encountering this educational virtual scenario vignette, the patient will be able to…)
Chronic case of AF^a^	Identify correct medication adherence practices Identify the correct frequency regimen of doctors’ appointments Identify correct dietary restrictions
Early-onset and paroxysmic AF	Identify initial onset of symptoms of AF Identify timely medical consultation practices Recognize the correct medicinal adherence procedure for the condition Identify the correct exercise intensity to manage the condition
AF with lifestyle- compounding factors to the disease (smoking, drinking, and unhealthy eating)	All educational objectives of “Chronic case of AF” Identify risks of high-impact complications Identify best practices for recovering from missed doses of medical treatment

^a^AF: atrial fibrillation.

Iterative brainstorming between the medical experts of all study centers will produce the detailed VP scenarios in the mobile digital platform (for details, refer to the previous section, *The Technical Architecture of the Mobile App*). After an internal review of these scenarios by the multinational expert panel, a selection would be made for the final case to be used. In that context, the team will choose to simply use 1 scenario in the multicenter multinational cohort trials. The most appropriate scenario would have the following characteristics: (1) it should be the most relevant—a significantly larger proportion of patients should be targeted; (2) it should be the most clinically impactful—it is important and useful for patients with AF to be aware of AF complications; and (3) it should be the most educationally important—both patients who fall within the parameters of the described case and those who do not would benefit from information and preventative knowledge of the impact of AF.

An acceptance rate of 75% by medical experts for each criterion for each scenario should be reached to be eligible for recruitment. Moreover, this patient education intervention will be based on techniques from the behavior change technique taxonomy—an international consensus for the reporting of behavior change interventions [28].

After this selection, a process of localization and adaptation to the specifics of each center (Greece, Ukraine, and Kazakhstan) would be conducted.

### Evaluation Design

#### Evaluation Instruments

For educational efficacy, a multiple-choice questionnaire (MCQ) for knowledge retention will be used (Textbox 2). The choice of questions was based on the European Society of Cardiology’s *Guidelines for Management of Atrial Fibrillation* [29]. This instrument will be translated for all participating centers in Greece, Ukraine, and Kazakhstan. The translation and evaluation of the translation will be performed by teams of bilingual experts. The instrument in its original version will be provided to bilingual persons in alternating language order and will be assessed accordingly. Scoring each questionnaire follows a simple process of allocating a numerical score equal to the number of choices in the MCQ to weigh each response for randomly selecting the correct question. For example, a question that has 4 possible responses in the MCQ will be scored 4 points if answered correctly, while a question that has 5 possible answers will be scored with 5 points if correctly answered.

**Questionnaire for knowledge retention on atrial fibrillation (the asterisk indicates the correct answer).**

**1. Atrial fibrillation:**

- Is the most common cardiac arrhythmia
- More often concerns older people, but can occur in any age
- May be related with thyroid disorders
- All of the above*

**2. The most common symptom of atrial fibrillation is:**

- Chest pain
- Palpitations*
- Dizziness
- Blurry vision

**3. How can atrial fibrillation be diagnosed?**

- Following an electrocardiogram evaluated by a cardiologist*
- By describing symptoms of the arrhythmia to the doctor
- By checking the indication ‘’arrhythmia’’ of the blood pressure device
- All of the above

**4. What is the most important complication a patient with atrial fibrillation not receiving treatment may suffer?**

- Fainting spells
- Myocardial infarction
- Lethal arrhythmia
- Ischemic stroke*

**5. Treatment with oral anticoagulants always mandates frequent blood tests:**

- True
- False*

**6. The patient with atrial fibrillation that visits a cardiologist:**

- Probably does not need oral anticoagulation to prevent stroke
- Always needs treatment to cure the arrhythmia
- Cardiologist? There is no need to see a doctor
- Might have to be admitted to the hospital at the time of Atrial Fibrillation diagnosis *

**7. When the patient with atrial fibrillation is being treated with an oral anticoagulant:**

- It is better to receive the reduced dose so as to avoid bleeding
- This is always stopped at 3 months, since the danger for stroke is gradually reduced
- It is fine if he/she occasionally misses a dose
- He/she has to adherently receive the right dose of the drug, as prescribed by his/her treating physician*

**8. The newer oral anticoagulants:**

- Are at least as safe and effective as warfarin in preventing stroke
- Have to be taken every day on a fixed schedule, so as to be effective
- Do not have important interactions with other drugs or food, in contrast to warfarin
- All of the above*

**9. Oral anticoagulants:**

- Are not necessary, in case the patient already receives other blood thinning medication, such as aspirin
- Are the most effective treatment in preventing stroke*
- Do not have any significant side effect
- All of the above

**10. A patient that is under warfarin:**

- Cannot switch to a newer oral anticoagulant if he/she has good anticoagulation control (international normalized ratio within the desired range)
- Can follow an unrestricted diet
- Can get advice from his doctor regarding treatment with a newer anticoagulant, so as there is no need for frequent blood tests*
- Always has good anticoagulation control (INR within the desired range) if he/she receives his medication in a fixed dose

#### Project Management

All project members will meet remotely every week to work through advances and challenges together and to provide methodological support to remain aligned with the protocol. Researchers will be hired and trained, regulate safety conditions, and oversee the data collection and analysis. The researchers will prepare the data collection tools and perform data collection, and ensure that the materials required are adequate and functional. The senior Greek PI (PDB) will coordinate the overall project.

### Ethical Considerations

Ethics approval has been obtained from the Bioethics committee of the Medical School of the Aristotle University of Thessaloniki (178/19-3-2020). This study will be conducted in line with the tenets of the Declaration of Helsinki, and no participant will be randomized unless written informed consent is available for that participant. Participants can withdraw from the trial at any time and will be informed and assured of such right. This study follows the principles of data protection and management described in the European Union’s General Data Protection Regulation.

### Confidentiality

All personal and collected data will be treated as confidential at all stages of the study and will be stored separately. The electronic data will be saved with metadata in university network drives, which are protected by usernames and passwords. The participant ID list that links the study participants and research data will be disposed of after 15 years. Institutions hold the ownership of registry data.

## Results

The trials will start in 2024 and are expected to end later that year or in early 2025 or when the needed sample size is achieved. The initial results are expected by March 2024.

To assess the results from the prepilot evaluation questionnaires, the Welch *t* test will be conducted. Of note, we decided to conduct the Welch *t* test because some of our sample sizes will be heavily unequal between intervention and control groups. The standard Student *t* test is robust to inhomogeneity of variance when sample sizes between cohorts are the same; however, this is not true for largely differing cohort sample sizes. The Welch *t* test does not assume homogeneity of variance and, hence, is robust to widely varying sample sizes [30,31].

## Discussion

### Expected Findings

The results of this study aim to demonstrate the efficacy of the VP educational modality in transferring knowledge in an impactful way so that it would be useful and retained by the learner. The rationale for this kind of expected efficacy may be attributed to various factors. The impact of information passed through narrative vehicles has been identified early on [32]. Additionally, the initiative that the learner has to guide the narrative through their choices facilitates engagement through 2 avenues: one avenue is the agency that the learner has over their narrative exploration, and the second one, dependent on the first but not identical, is the ability to direct the narrative toward educational needs that the learner may have. These factors are compounded by the immediacy and ease of access that the mobile delivery platform offers, which can create an engaging and user-friendly experience that may amplify the educational impact.

This work will focus on knowledge retention and efficacy and not on perceived changes in quality of life. The impact of AF itself in the quality of life of patients is well documented with a validated questionnaire that has been available since the last decade [33]. A cursory search with the keyword “AFEQT” revealed more than 550 references on Google Scholar, including several reviews (for characteristic examples, see Kotecha et al [34] and Parameswaran et al [35]).

On the other hand, there is a significant body of literature that has identified the perceived impact of impactful patient education in their risks for serious complications and quality of life. AF, when first diagnosed, is an overwhelming situation for the patient, and reliable information is one of the first requirements for alleviating the initial possible shock [36,37]. Furthermore, lack of knowledge leads patients with AF to have significantly skewed perceptions about the importance of their condition and the true risks that stem from this potentially life-threatening, possibly chronic, condition.

Multiple studies have demonstrated that patients do not identify the possibility of stroke as an acute complication of AF; a lot of them cannot even identify their arrhythmias as AF, and they do not recognize AF as life-threatening even though they receive verbal or printed information about their condition [38,39].

In that context, it is very important to constantly explore information and educational avenues that are impactful and engaging for patients who need reliable and immediately absorbable information. This study aims to demonstrate that in this very important aspect of knowledge retention, the approach of narrative virtual scenarios is one that could provide a distinct impact advantage over the conventional paper-based or verbal information to the patients.

### Limitations

The study’s core limitation lies with the knowledge retention questionnaire. This is not a validated instrument. While we were aware of a formal validated instrument (the Jessa Atrial fibrillation Knowledge Questionnaire [40]), the knowledge spectrum that it covers is far wider than what our VP vignette may cover. Given that verbal and printed conventional information covers all relevant material, while our VP may cover specific critical topics related to AF, using a wide instrument such as the Jessa Atrial fibrillation Knowledge Questionnaire would run the risk to evaluate knowledge retention gaps that the VP vignette cannot cover. While a counterargument can be made that we are thus narrowing the evaluation scope to the strong points of our VP resource, the concise focus of the VP vignette is itself an argument for implementing it as a more effective educational tool in personalized and focused endeavors for patient empowerment. As a follow through too, we aim to address the second weakness of our study, which is the lack of a qualitative exploration about the acceptance of patients regarding the electronic medium of mobile devices and the modality of VPs in comparison to other existing modalities such as video demonstration or even gamified virtual environments.

Even given these limitations, however, this study can provide evidence for the comparatively better efficacy of the VP modality in mobile media for impactful and effective information for and empowerment of patients with AF. This study would be the first evidence-based step to initiate this process toward better informing and subsequent empowerment of patients with regard to the management of their disease.

### Conclusions

This project can generate new knowledge and relevant results for a deployed VP regimen for the education and engagement of patients with AF on crucial topics regarding their condition. A 3-center pseudorandomized controlled trial could add data to the evidence regarding the effects of interventions using VP resources in outreach and training endeavors for empowering patients with AF.

## Abbreviations

AF: atrial fibrillation
DEEP-RAFT: Doctors’ Education, Empowerment of Patients, Regarding Atrial Fibrillation and Venous Thromboembolism
HCP: health care professional
MCQ: multiple-choice questionnaire
mHealth: mobile health
NOAC: non–vitamin K oral coagulant
OAC: oral anticoagulant
RCT: randomized controlled trial
VKA: vitamin K antagonist
VP: virtual patient
